# Kinetic model of biomass hydrolysis by a polysulfone membrane with chemically linked acidic ionic liquids *via* catalytic reactor†

**DOI:** 10.1039/c8ra00658j

**Published:** 2018-03-27

**Authors:** Peng Lu, Yong Cao, Xiaolan Wang

**Affiliations:** School of Chemistry and Chemical Engineering, Beijing Institute of Technology Beijing 102488 P. R. China penglu@bit.edu.cn

## Abstract

A novel catalytic membrane was prepared from polysulfone powders covalently linked with acidic ionic liquids (PSF-ILs), as a heterogeneous catalyst to produce reducing sugar for biomass inulin hydrolysis in a catalytic membrane reactor. However, a complete kinetic model describing the relationship between catalyst physical properties and hydrolysis performance is lacking. The present work attempts to build a hydrolysis kinetic model for the relationship between the conversion and the structure of the PSF-ILs membrane (such as membrane thickness, pore size, porosity and specific surface area). The reaction parameters, such as the reaction time, reaction temperature as well as the nature of the catalyst were of crucial importance in terms of optimal conversion of the biomass inulin to reducing sugar or platform chemicals. The results showed that the structure of the PSF-ILs membrane has a significant effect on its catalytic performance. The PSF-ILs membrane with the best catalytic performance was selected and the maximum TRS yields were up to 100% after two rounds of inulin hydrolysis. The conversions obtained from the established model are in good agreement with the experimental data. Understanding the structure–property relationship of the PSF-ILs membrane will be helpful in designing the physical structure of the membrane to improve its catalytic activity and reusability. Therefore, it is a type of green catalyst with potential application prospects in many catalysis fields.

## Abstract 2

The PSF-ILs membrane exhibits higher catalytic performance and a kinetic model between conversion and membrane structure was established successfully.

## Graphical abstract

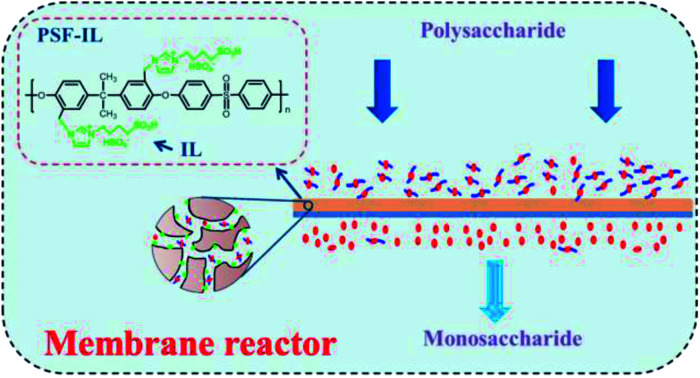

## 1. Introduction

In recent years, the growing demand for energy has created the need for the development of sustainable technologies based on renewable raw materials.^1^ Cellulosic biomass, such as corn stover, forest products residue, and energy crops, has been identified as an important source for fuel alternatives and value-added chemicals.^2^ Among others, the Jerusalem artichoke, as an energy plant, has the capability of being processed into biofuel without competing with a major food source. As a biofuel, ethanol is by far the mostly widely used worldwide for the transportation sector, since it has a higher octane number, broader flammability limits, and higher flame speeds and heats of vaporization.^3^ Therefore, the highly-efficient conversion of biomass polysaccharide to biofuel begins with a hydrolysis process for reducing sugars production, which has significant benefits for subsequent fermentation. Conventionally, the hydrolysis method was carried out using acid^4^ or enzyme^5^ as catalysts. However, several disadvantages of these catalysts made this process inefficient and uneconomical. Inorganic acids could cause the problems of equipment corrosion, severe environmental pollution and generally expensive for commercial use.^6^ As for enzyme, it is difficult to recycle and low efficiency as a result of the toxic side effects of the microorganism.^7^ Thus, developing environmental friendly catalysts with wide industrial application for producing fermentable sugar from biomass is an urgent task.

Ionic liquids (ILs) are relatively new class of environment-friendly catalyst that has evolved as a hot spot in the field of biomass hydrolysis. Amarasekara *et al.*^8^ applied the ILs (1-(1-propylsulfonic)-3-methyl-imidazolium chloride) catalyzed hydrolysis of Sigmacell cellulose with the total reducing sugar of 62% and immobilized this kind of ILs to fabricate a silica catalyst for cellulose hydrolysis, a maximum TRS yield of 67% was obtained at 70 °C for 360 min. Furthermore, some influential studies towards immobilizing of imidazolium-based alkoxide ILs on poly(ether sulfone) materials also have been done and have demonstrated its effectiveness to improve the conductivity of the materials.^9,10^ The immobilization of ILs promoted the recovery of the catalyst from hydrolysates and avoided the potential hazards of homogenous ILs to the environment.

Kinetic modeling may be regarded as an important step in developing a biomass hydrolysis process, since dynamic models could accurately describe the experimental data and control the reaction process.^11^ Moreover, the established model could help us to further understand the hydrolysis process in a deep insight. The hydrolysis kinetics of inulin using the imidazole-based acidic ILs as catalyst was successfully established by Zhao *et al.*^12^ And the modeling results showed that the reaction rate constant was associated with reaction condition, such as catalyst concentration and hydrolysis temperature. Shah *et al.*^13^ established a kinetic model for the esterification reaction in the catalytic membrane reactor and predicted the catalytic behavior of membrane catalyst as a function of catalyst concentration and reaction temperature. In the previous studies,^14,15^ various kinetic models have been proposed to describe the relationship between the conversion and the operating reaction condition.

Actually, for heterogeneous catalytic system, the reaction conversion is related not only to the operating conditions, but also the physical properties of the catalysts (such as pore size, porosity and specific surface area *et al.*). Kamaruddin *et al.*^16^ studied the kinetic process of the transesterification using lipase as catalyst in a packed bed reactor. And the results revealed that the optimum packed bed height is 9 cm with the yield of 75%. In addition, the kinetic model of esterification was established with cation-exchange resin as a heterogeneous catalyst.^17^ It was found that increasing bed height would increase the retention time, resulting in higher production conversion. However, only a few kinetic reports are available for the ILs hydrolysis of biomass to reducing sugar in heterogeneous catalytic system.

The objective of the present work is to prepare a catalytic membrane from PSF-ILs powders as a heterogeneous catalyst to produce reducing sugar in a catalytic membrane reactor and to research the regulation of membrane morphology and its catalytic performance. Meanwhile, based on the analysis of the literature, a complete kinetic model describing the relationship between catalyst physical properties and hydrolysis performance in heterogeneous system is lacking. Thus, a mathematical model would be developed to elucidate the relationships between the catalytic membrane structure and hydrolysis performance of the membrane. The membrane thickness, pore size, surface area and porosity of the catalytic membranes were taken into account in the kinetic study. Furthermore, the established kinetic model could be also applied to predict the biomass hydrolysis in wider ranges of experimental conditions. The obtained PSF-ILs membrane has a great potential application for preparing fermentable reducing sugar for ethanol production.

## 2. Materials and methods

### 2.1. Chemicals

Polysulfone (PSF) (Udel 3500, *M*_w_ = 30 000) was used as membrane material and was purchased from Hangzhou Group Co., Ltd. (Hangzhou, China); Inulin (polysaccharide: 94.2 initial reducing sugar: 3%, moisture and ash: 2.8%) was purchased from Gansu Likang Nutrition Food Corporation (Lanzhou, China); imidazole, 1,4-butylesultone and zinc chloride were purchased from Aladdin Chemical Company (Beijing, China); *N*,*N*-dimetbylformamide (DMF), *N*-methyl-2-pyrrolidone (PVP), PEG600 and ethanol were used as analytical reagents and were purchased from Tianjin Fuchen Chemical Reagent Factory (Tianjin, China); all chemicals used in this study were of analytical grade and used without purification.

### 2.2. Synthesis of ILs modified polysulfone (PSF-ILs) powders

The membrane used in this work was the PSF membrane covalently modified by acidic ILs. The PSF-ILs powder was synthesized in four steps. In the first step, the PSF-Cl powder was prepared using the method presented by Zhang.^18^ 3.0 g PSF was completely dissolved in 150 mL dichloromethane with a three necked round-bottomed flask at room temperature, then 0.3 g anhydrous zinc chloride and 7 mL chloromethyl ether were added and stirred at 50 °C for 12 h. After the reaction completed, the reaction mixture was poured into 500 mL ethanol and mass white solid was formed. The resulting product was filtered and washed with ethanol and dried under vacuum at 50 °C for 24 h. In the second step, 3 g PSF-Cl and 0.39 g imidazole was added in 150 mL dichloromethane. The mixture was stirred at 50 °C for 10 h, and then disposed using aforementioned method to get PSF-Im powders. In the third step, the dried PSF-Im powders with 0.64 g 1,4-butane sultone were introduced in 150 mL dichloromethane and stirred at 50 °C for 8 h. After the same treatment, the PSF-Im-SO_3_H powder was obtained. In the last step, the PSF-Im-SO_3_H powders with 120 mL dichloromethane were put in the 250 mL flask. After complete dissolution, an equimolar concentrated sulfuric acid was added slowly in ice water bath. Then the final mixture was slowly heated to 50 °C for 12 h. Finally, the product was precipitated and washed with large quantities of ethanol and deionized water, and the pulverous PSF-ILs catalyst was dried under vacuum at 50 °C for 12 h. The detailed characterization results of the Fourier Transform Infrared and Nuclear Magnetic Resonance were showed in our previous study (showed in ESI Material†).

The degree of chloromethylation (DCM) of PSF, defined as the average number chlormethyl groups per repeat unit of PSF, was used to evaluate the immobilization amount of catalyst. DCM of chloromethylated polymers is an important parameter, which determines the catalytic performance of the PSF-IL to a great extent. DCM can be calculated by the following equation,1
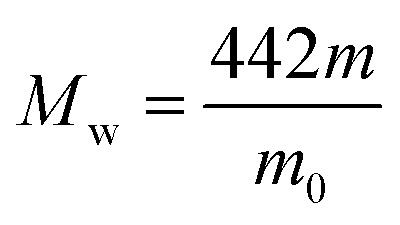
2
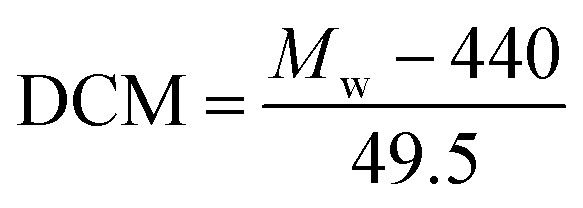
where *m*_0_ and *m* were the mass of PSF and chloromethylated PSF (CMPSF) powders, respectively. *M*_w_ is the relative molecular mass of CMPSF. 440 is the molecular weight of PSF repeat unit, 440 is the molecular weight of PSF repeat unit subtracting two hydrogen atoms on the phenyl ring and 49.5 is the molecular weight of chloromethyl group (–CH_2_Cl).

### 2.3. Viscosity test

The viscosity of the casting solution was one of the most important parameters that affected the phase separation behaviors. Viscosities of dope solutions prepared from the different coagulation bath temperature with PSF-ILs concentration of 17 wt% were investigated using a viscometer (LVDV-II+P, Brookfield, United States) with S41 spindle. For each test, a 2 mL sample was used and the measurements were performed in duplicate. The temperature of the sample was maintained *via* an external temperature controller. The viscosity results of the tested casting solution with different external temperature were investigated. As shown in Table 1, it is found that the viscosity of the tested casting solution had a decrease as the coagulation bath temperature increased.

**Table 1:** Viscosity of PSF-ILs solutions with different coagulation bath temperature

Temperature	30	35	37	40	43	45	50	55	60
Viscosity/cP	1332.5	1260.3	1195.7	1142.2	1066.5	998.1	851.7	790.6	700.1

### 2.4. Membrane preparation

Membranes were prepared using the immersion precipitation technology.^19^ A certain amount of PSF-ILs powders were dissolved in dimethyl formamide (DMF) at room temperature, followed the additive polyvinylpyrrolidone (PVP) and polyethylene glycol 600 (PEG600) were added. After complete dissolution, the homogeneous polymer solution was left in vacuum oven to disengage air bubbles before membrane casting. The clear solution was casted onto a clean, smooth glass plate, and the thickness of the nascent membranes was controlled by a casting machine, and then the casting solution were prepared by a steel casting knife with a speed of 3.6 m min^−1^. The casting solution was subsequently immersed in non-solvent exchange bath for 24 h. When polymer solution was added to the non-solvent, the exchange mass transfer between the solvent and the non-solvent occurred immediately, and induced phase inversion, in which polymer-rich phase and polymer-poor phase were formed simultaneously, followed by solidification of the polymer-rich phase. As a result, PSF-ILs membranes can be formed. The membranes were air dried to remove residual solvent from the pores, and then their properties were tested.

### 2.5. Membrane characterization

#### 2.5.1. SEM

Field emission scanning electron microscopy (JSM-7401, LEI, Japan; JSM-7500F, JEOL, Japan) was used to obtain the morphologies of the tested membranes. A dry PSF-ILs membrane was frozen and fractured in liquid nitrogen to measure the cross section.

#### 2.5.2. Contact angle (CA)

An optical contact angle measurement system (DataPhysics Instruments GmbH, Filderstadt, Germany) was used to measure CA of the prepared membranes. A droplet with volume of 0.2 μL deionized water with a rate of 5 μL s^−1^ automatically controlled by CA analyzer was measured on the tested membrane samples surface at room temperature. Multiple CAs values were measured and the average values were obtained from at least five CAs at different locations on the tested membranes.

#### 2.5.3. Pore size distribution and porosity

The pore size distributions of the membranes were measured by a flow aperture instrument (3H-2000PB, Beishide, China). A piece of membrane samples with diameters of 25 mm were prepared. Before the measurement, the membrane was fully infiltrated with a Porofil liquid (Perfluor-oether, surface tension 19 mN m^−1^). A control software was used to calculate the pore size of the samples.

The porosity is defined as the volume of pores divided by the total volume of the membrane. Each dry membrane was cut into a circle with a diameter of 25 mm. The dry membrane sample was first weighted and then immersed in *n*-butyl alcohol for about 24 h. Finally, the wet membrane was weighted as soon as the superficial solvent on the membrane was removed with dry filter paper. The porosity was calculated as follows.^20^3
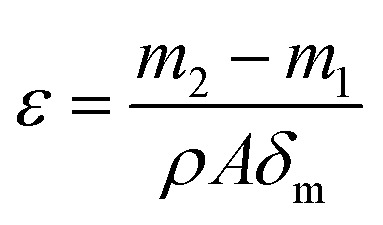
where *ε* is the (%) porosity of the membrane, *m*_1_ and *m*_2_ are the weights of dry membrane and wet membrane (g), respectively. *ρ* is density of *n*-butyl alcohol (0.801 g cm^−3^), *A* is the effective area of the membrane (cm^2^), and *δ*_m_ is the thickness of the membrane (cm). All reported porosity is averages of three different membrane samples in our experiments.

#### 2.5.4. Pure water flux and rejection

The membrane holder has a 2.5 cm outer diameter and an effective filtration area of 10^−2^ cm^2^. The pure water flux was determined with deionized water using 0.1 MPa of N_2_ pressure as the driving force at ambient temperature of 20 ± 2 °C. The weight of pure water was then recorded by an electronic balance every 30 s for 10 min. The pure water flux (*J*) was calculated by the following equation, and the values were averaged:4
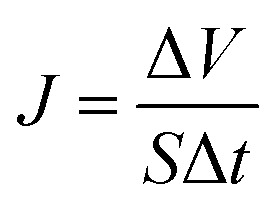
where Δ*V* is the permeate volume, *S* is the surface area of the membrane (m^2^), and Δ*t* is the time needed to collect the permeate.

A concentration of 0.1 g L^−1^ of bovine serum albumin (BSA) solution was used to characterize the membrane rejection at 0.1 MPa and 25 °C. A calibration curve of the absorbance against the BSA concentration was constructed. The BSA concentration was measured at a wavelength of 280 nm with a UV spectrophotometer (UV4802, Unico, United States). The rejection (*R*) was calculated as follows:5
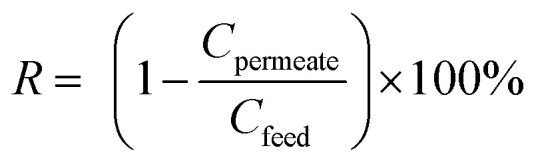
where *R* is the (%) protein rejection ratio of the membrane, *C*_permeate_ and *C*_feed_ are the concentration of solute in the permeate and feed (g L^−1^), respectively. All reported water fluxes and BSA rejections are averages of three different membrane samples in our experiments.

### 2.6. Inulin hydrolysis and total reducing sugar (TRS) assay

The hydrolysis experiments were conducted in a self-made stainless steel reactor with the fixed reaction conditions. PSF-ILs membrane with diameter of 60 mm was installed on the flat membrane reactor, then 100 mL 20% (%, the weight ratio of the inulin to water) inulin aqueous solution was heated to fixed temperature and immediately added to the membrane reactor. Keeping the solution in the set temperature, inulin aqueous solution could be permeated to the hydrolysate collector across the membrane at a fixed flow rate using a peristaltic pump to supply circulation power. At the same time, 15 μL sample from the hydrolysate was transferred into a vial for reducing sugar assay at set intervals. The assay method of total reducing sugar (TRS) was determined by 3,4-dinitrosalicylic acid (DNS) method.^21^

### 2.7. Kinetic model

**Fig. 1:**
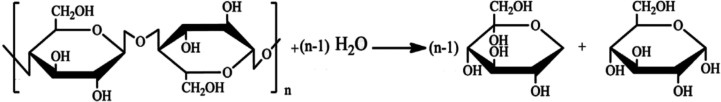
Hydrolysis reaction of inulin polysaccharide.

Inulin is a kind of polysaccharide compounds with different degree of polymerization. It is a series of parallel reactions with different degree of polymerization in hydrolysis reaction. Thus, it is difficult to detect the concentrations of polysaccharide. The degree of polysaccharides hydrolysis could be tested by measuring the concentration of reducing sugar produced, the concentrations of reactants and products were related to each other by a mass balance based on the stoichiometry of the reaction scheme. The hydrolysis reaction of inulin polysaccharide for producing reducing sugars was shown in Fig. 1, where *n* represents the polymerization degree of polysaccharides.

During the process of inulin hydrolysis, the production of *n* − 1 fructose molecules and 1 glucose molecules were obtained. Before the kinetic model was established, it is assumed that the concentration of the water medium is constant and the molecular weight of the polysaccharide molecules is average. At the same time, the reaction rate was proportional to the concentration of polysaccharide, which accorded with the first-order reaction law. Therefore, the first-order kinetic equation is used to describe the reaction process:6
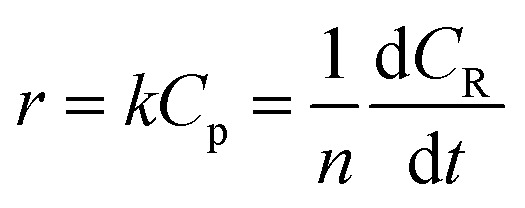


Among them, *r* is the reaction rate, *k* is the first-order reaction rate constant, *t* is the reaction time, *n* is the average degree of polymerization (*n* = 2–60) of inulin polysaccharide, *C*_R_, *C*_p_ is the concentration of reducing sugar and polysaccharide concentration (mol L^−1^). Based on the relationship between polysaccharide and reducing sugar, the conversion rate of *R* can be expressed as7
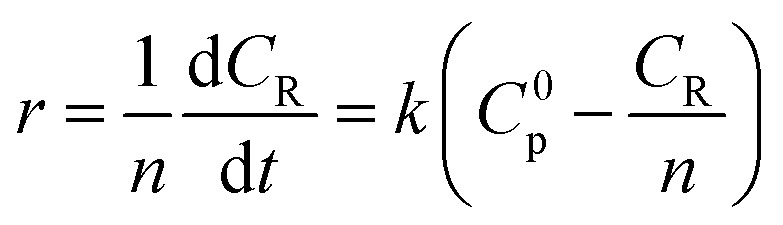
where *C*^0^_p_ is the initial concentration of polysaccharides in mol L^−1^.

In the previous study,^12^ the proposed kinetic model of inulin hydrolysis catalyzed by homogeneous ILs was established and it successfully predicted the inulin hydrolysis in wider ranges of experimental conditions. In this work, the hydrolysis kinetics of PSF-ILs membrane was also studied in inulin hydrolysis system. Due to the catalytic mechanism of inulin hydrolysis catalyzed by PSF-ILs membrane is consistent with that by the homogeneous ILs, the proposed kinetic model may also be employed to describe the inulin hydrolysis by the former. The hydrolysis kinetics followed the typical first order model with hydrolysis reaction.^22^ Hydrolysis ratio (*R*) can be obtained in the following equation,^12^8
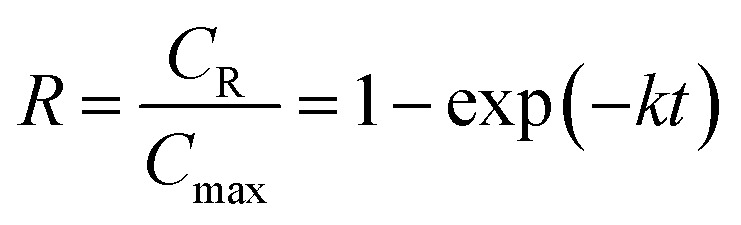
where *C*_R_ is the reducing sugars concentration, and *C*_max_ is the final reducing sugars concentration when inulin is hydrolyzed completely (mol L^−1^). In the inulin hydrolysis system catalyzed by PSF-ILs membrane, *t* was the residence time in the membrane, in this study, the retention time refers to the time required by the substrate solution (as a particle) from the beginning of entering the reactor to the outflow reactor.

The kinetic constant *k* is a function of absolute temperature according to the Arrhenius expression as follows9
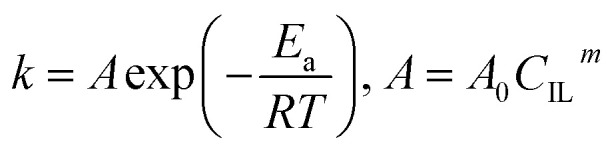
where *E*_a_ is the activation energy (kJ mol^−1^), *R* is the molar gas constant (8.314 is10^−3^ kJ mol^−1^ K^−1^), *T* is the temperature (K), *A* is the pre-exponential factor (min^−1^) and was expressed by the corresponding literature.^23^*A*_0_ is the pre-exponential parameter for inulin hydrolysis (min^−1^), *m* is the ILs concentration exponent for the rate constant *k*. In addition, it is reported that activated energy and pre-exponential factor are related to the concentration of catalyst (*C*_IL_), respectively.^12,23–25^ Zhao *et al.* established kinetic model for the biomass hydrolysis in homogeneous catalytic system and considered that the activation energy and the pre-exponential factors are dependent on catalyst concentration.^12^ Meanwhile, the study about kinetic characterization of biomass hydrolysis by dilute sulfuric acid reported that reaction steps are irreversible and follow a first-order kinetic dependence on catalyst concentration, thus, activated energy and pre-exponential factor are related to the concentration of catalyst.^24,25^ In the process of inulin hydrolysis, the activation energy of the whole process cannot be kept constant because of the competition between the hydrolysis mechanism of ILs. Thus, *E*_a_ could be expressed as follows:10
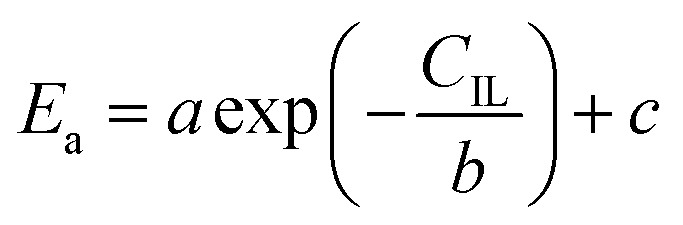
where *a*, *b* and *c* are constants.

Based on the above theoretical analysis, in order to further analyze the hydrolysis process and understand the relationship between sponge-like PSF-ILs membrane structure parameters (membrane thickness, porosity, pore size, *etc.*) and catalytic performance, the kinetics model was built on the following assumption according to the Barrett–Joyner–Halenda method:^26^ (i) the cross section of the PSF-ILs membrane consists of numerous solid cylindrical columns. (ii) The external specific surface area of the PSF-ILs membrane is neglected due to the internal specific surface area far higher than external specific surface area. Taking into account the actual sponge-like structure of the PSF-ILs membrane, the correction coefficient is used to correct the model. The modeling process is described as following:

The volume and surface area of a single cylindrical channel were expressed as the following equation, respectively:11
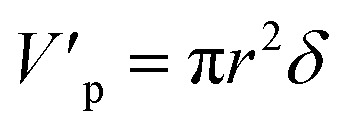
12
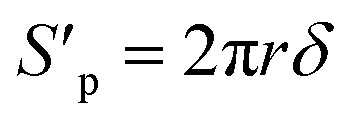
where *δ* is the membrane thickness, *r* is the radius of the single cylindrical channel.

The actual volume of the inner pore of the PSF-ILs membrane can be written as

13 *V*_p_ = *εV*_m_ = *εSδ*

where *V*_m_ is the actual volume of the whole membrane, *ε* is porosity, *S* is surface area of PSF-ILs membrane.

Since the number of cylinders is constant, thus, combing eqn (11)–(13), the actual internal specific surface area of the PSF-ILs membrane could be expressed as14
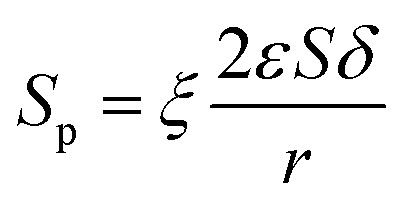
where *S*_p_ is the internal specific surface area of the PSF-ILs membrane, *ξ* is the tortuosity.

In the process of catalysis, the inulin hydrolysis is mainly catalyzed by the catalyst which immobilized on the specific surface area of the membrane. The relationship of the concentration of catalyst and the internal specific surface area of the PSF-ILs membrane could be expressed as

15 *C*_IL_ = *φyS*_p_

Where *y* is loading amount of ILs, *φ* is the proportionality coefficient.

Combing eqn (10), (14) and (15), the activation energy and membrane structure parameters could be written as16
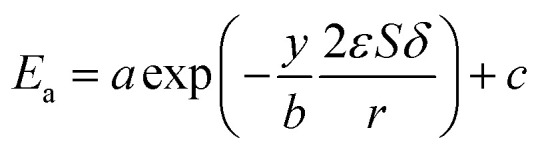
where *b* is constant.

## 3. Results and discussion

### 3.1. SEM analysis

**Fig. 2:**
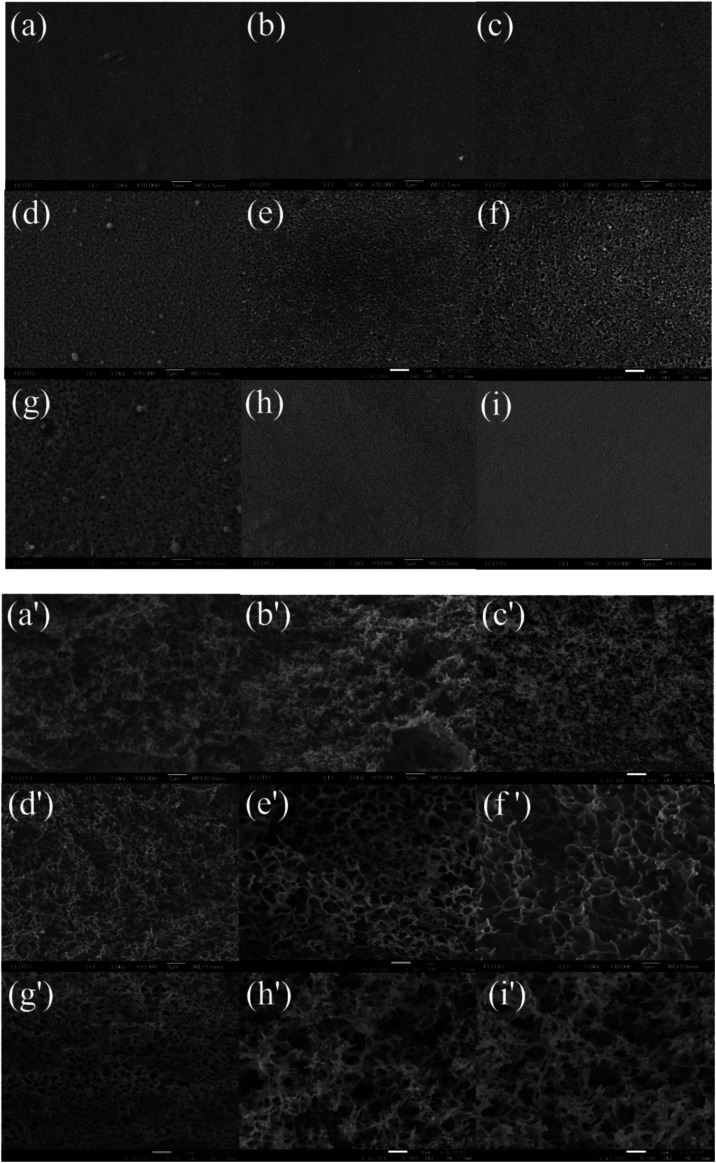
SEM micrographs showing the membrane structure obtained at different coagulation bath temperatures: surface (10 000×) and cross section (10 000×): (a and a') 30 °C, (b and b') 35 °C, (c and c') 37 °C, (d and d') 40 °C, (e and e') 43 °C, (f and f') 45 °C, (g and g') 50 °C, (h and h') 55 °C, (i and i') 60 °C. The PSF-ILs concentration was 17%.

Surface and cross-sectional SEM images of the membranes prepared from different coagulation bath temperature, including 30, 35, 37, 40, 43, 45, 50, 55 and 60 °C, were shown in Fig. 2, respectively. When the casting solution was quickly immersed in non-solvent, the mass transfer of solvent and non-solvent almost simultaneously took place between the surface and inner of nascent membrane. The results showed that the coagulation bath temperature had a strong influence on the physical morphology of the prepared membrane. This behavior can be explained by the rate of the demixing process.

When the temperature of coagulation bath was lower than 40 °C, demixing was delayed, precipitation was slowly, thus it took much longer for membrane to form. As a result, membrane with a relatively dense top layer and sponge-like substructure were obtained. When the temperature of coagulation bath was 40 °C and even higher, increased the temperature of coagulation bath would cause a rise in diffusional flow rate between solvent and non-solvent and polymer chains became more flexible, in which faster demixing taken place and the loose porous cross-section structure was generally formed. Meanwhile, increasing the temperature of coagulation bath caused a decrease in surface pore size due to the fast solvent–nonsolvent exchange rate on the interface between casting solution and non-solvent.

### 3.2. Pore size

**Fig. 3:**
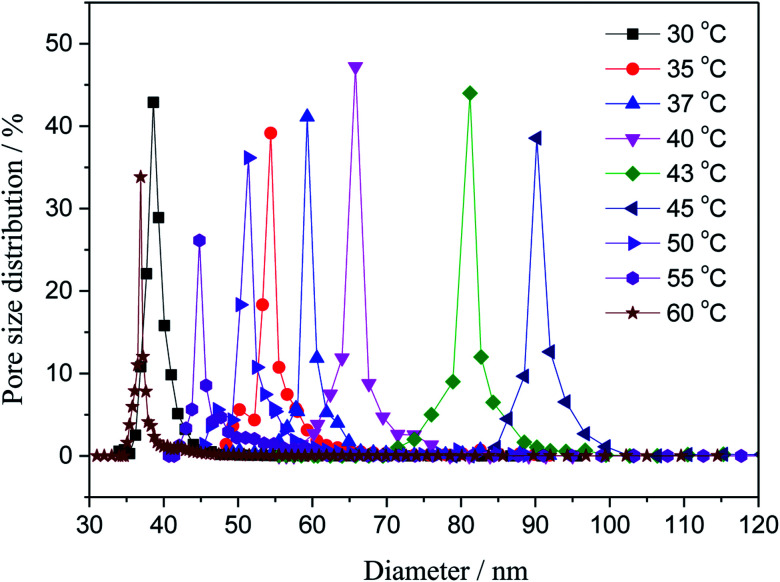
Variation of the pore size with the coagulation bath temperature at a constant PSF-ILs concentration of 17 wt%.

The pore distributions of the fabricated membranes with different coagulating bath temperature were investigated. As shown in Fig. 3. The increase in coagulation bath temperature intensively increased the mutual diffusivity between the nonsolvent and the solvent in the casting solution during the solidification process, leading to the formation of larger pore in the surface of the nascent membrane. The maximum pore size is 92 nm with coagulation bath temperature of 45 °C. However, when the coagulation bath temperature is greater than 45 °C, the most probable pore size was apparently minished, and it reduced diameter to 35 nm at the coagulation bath temperature of 60 °C. This behavior can be primarily explained by the different rate of the demixing process.^27,28^ The surface would be solidified in the membrane–bath interface prior to the inner of the nascent membrane, owing to the diffusion of non-solvent is faster than outflow of solvent molecules at the higher temperature.

### 3.3. CA

**Fig. 4:**
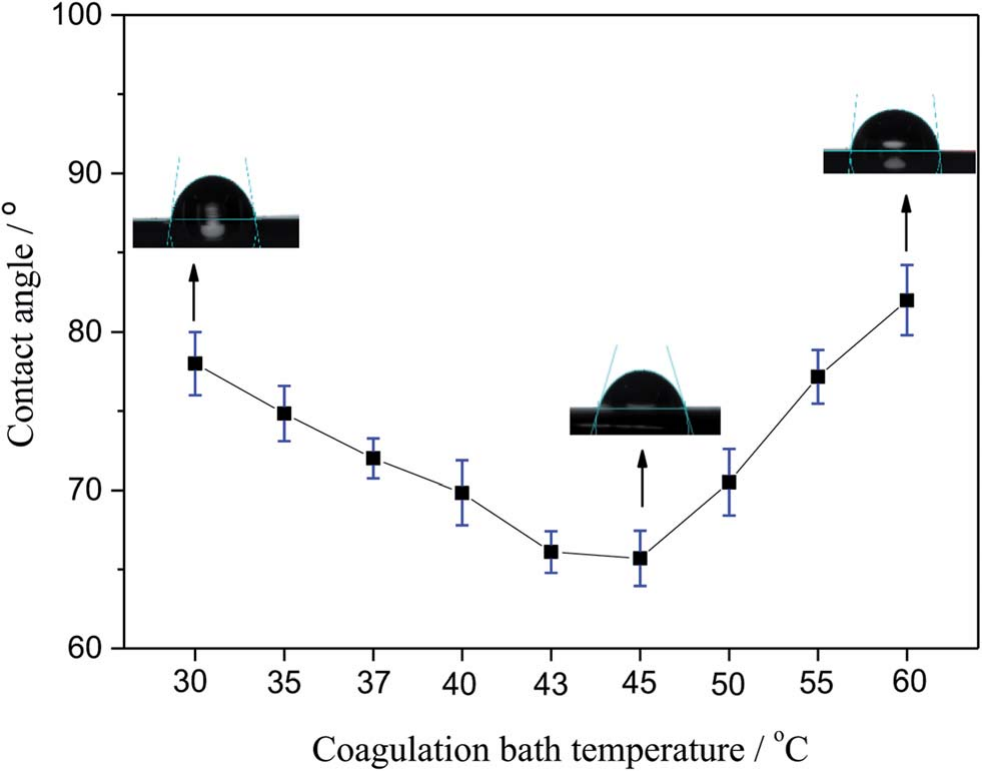
Variation of the contact angle with the coagulation bath temperature.

The CA of membranes prepared with different coagulating bath temperature under the same PSF-ILs concentration of 17 wt% were shown in Fig. 4. The CA value tended to decrease as the coagulation bath temperature increased from 30 to 45 °C. However, CA was grown gradually with the increase in coagulation bath temperature when that became higher than 45 °C. This behavior can be explained in terms of the results of pore size distribution. The presence of the ILs molecules has led to the macroporous formation in the surface of membrane. Thus, when the pore size of prepared membrane was larger, it was more sensitive to the pure water, thus resulting in low CA value.^29^

### 3.4. Porosity

**Fig. 5:**
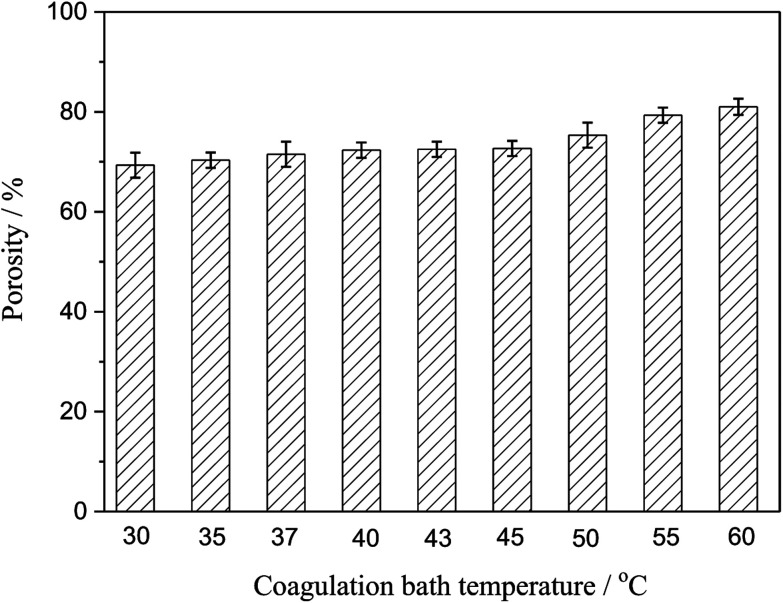
Variation of the porosity of the PSF-ILs membrane with the coagulation bath temperature at a constant PSF-ILs concentration of 17 wt%.

Porosity was also an important parameter of the membranes. Fig. 5 presented the porosity of the PSF-ILs membrane, which gently grown with the increase coagulation bath temperature at a certain PSF-ILs concentration. This phenomenon could also be explained from microstructure of the membranes shown in Fig. 2, where the increase in the porosity was mainly related to the connected channel structure. On the other hand, the decrease in the viscosity of the casting solution increases the movement of the polymer molecules chains during the phase separation. At that moment, membrane formation carried on in a faster phase separation process.

### 3.5. Water flux and rejection measurements

**Fig. 6:**
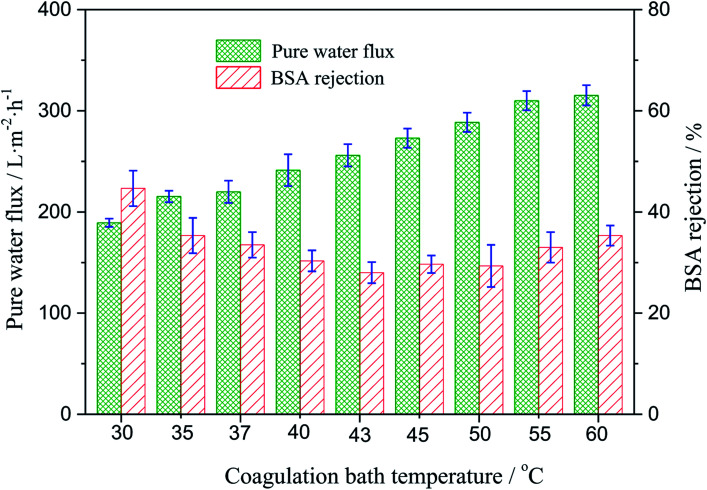
Variation of the water flux and BSA rejection of the PSF-ILs membrane with the coagulation bath temperature at a constant PSF-ILs concentration of 17%.

The effect of the coagulating bath temperature under the same PSF-ILs concentration of 17 wt% on pure water flux and rejection had been studied as well. As shown in Fig. 6, the pure water flux increased from 189.3 to 315.3 L m^−2^ h with an increase in the coagulation bath temperature from 30 to 60 °C. The reason for the increase in the water flux was the speed of phase exchange. The higher coagulation bath temperature accelerated the speed of phase exchange and induced the formation of the nascent membrane, resulting in an increased pore density. The effect of the coagulation bath temperature on the rejection was resulted from the variation trend in the pore size of the tested membranes.

### 3.6. Kinetics study

**Fig. 7:**
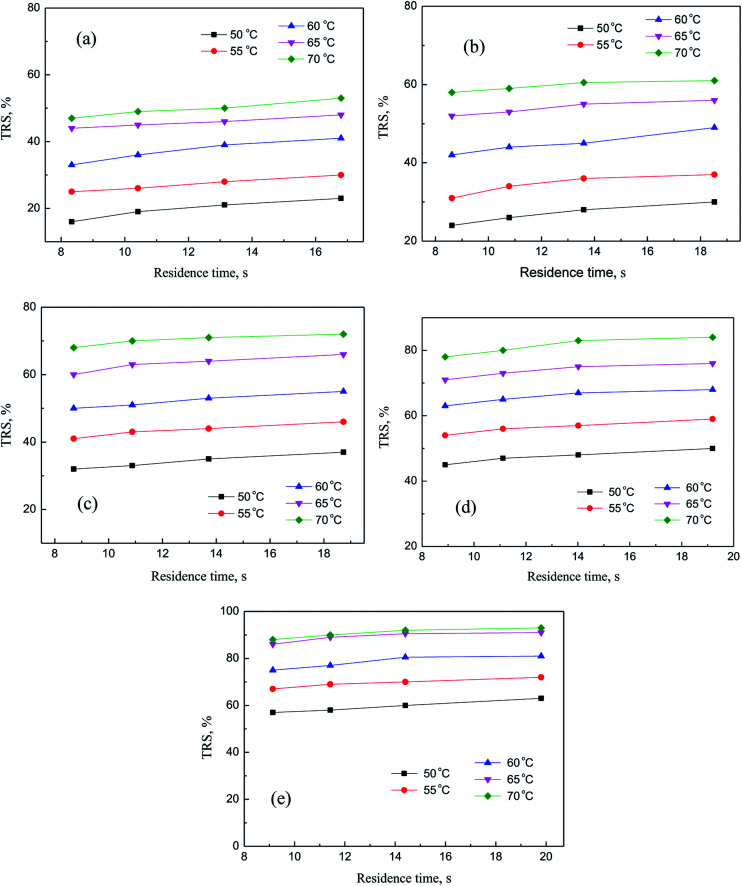
Catalytic performance of PSF-IL membrane under different residence time through membrane. DCM: (a) 0.45, (b) 0.75, (c) 1.08, (d) 1.42, (e) 1.71.

In this section, independent experiments of membrane hydrolysis and reaction kinetic were carried out to estimate modeling parameters. Firstly, the effect of different residence time on the catalytic performance of PSF-ILs membrane was studied. It can be seen from Fig. 7(a–e) that the conversion rate of inulin increased with the increase of reaction temperature and catalyst loading amount at the same reaction time. In addition, the conversion rate of inulin increased with the increase of membrane flux at the same reaction temperature. It is mainly because of the increase of the residence time of the inulin solution in the membrane, the contact time between the glycosidic bond and the active site of the catalyst is relatively prolonged, which is more favorable for the catalytic reaction.

The activation energy and pre-exponential factor were calculated by eqn (9) and (10). The kinetic parameters of inulin hydrolysis catalyzed by PSF-ILs membrane were shown in Table 2. The calculated reaction rate constant *k*, reaction activation energy *E*_a_, and pre-exponential factor *A* (as shown in Table 2) and the corresponding membrane structure parameters (as shown in Table 3) are brought into eqn (16). Meanwhile, the effects of membrane thickness, porosity and pore size on catalytic performance of membrane that was constructed as a reactor were investigated in our previous work. The TRS increases gradually with increasing the membrane thickness, porosity and pore size of the PSF-ILs membrane. The detailed research results were showed in our previous study (showed in ESI Material†).

**Table 2:** The hydrolysis kinetics parameters with membrane flux of 49.4 L m^−2^ h

DCM	*k*					*E*_a_ (kJ mol^−1^)	ln *A*	*R* ^2^
	50 °C	55 °C	60 °C	65 °C	70 °C			
0.45	0.0189	0.0250	0.0376	0.0469	0.0528	49.59	14.52	0.9597
0.75	0.0242	0.0328	0.0440	0.0587	0.0683	49.14	14.59	0.9877
1.08	0.0311	0.0444	0.0550	0.0744	0.0902	48.88	14.76	0.9902
1.42	0.0435	0.0602	0.0791	0.0989	0.1264	48.55	14.96	0.9956
1.71	0.0662	0.0836	0.1135	0.1634	0.1753	48.34	15.28	0.9653

**Table 3:** The structure parameters of PSF-IL membrane

DCM	Loading/mol g	Weight g	Porosity/*ε* %	Pore size/*r* nm	Thickness/*δ* μm
0.45	0.959 × 10^−3^	0.2078	76.9	86	240
0.75	1.102 × 10^−3^	0.2150	77.1	83	242
1.08	1.242 × 10^−3^	0.2304	77.9	84	243
1.42	1.381 × 10^−3^	0.2441	78.5	83	245
1.71	1.523 × 10^−3^	0.2605	79.7	80	248

**Fig. 8:**
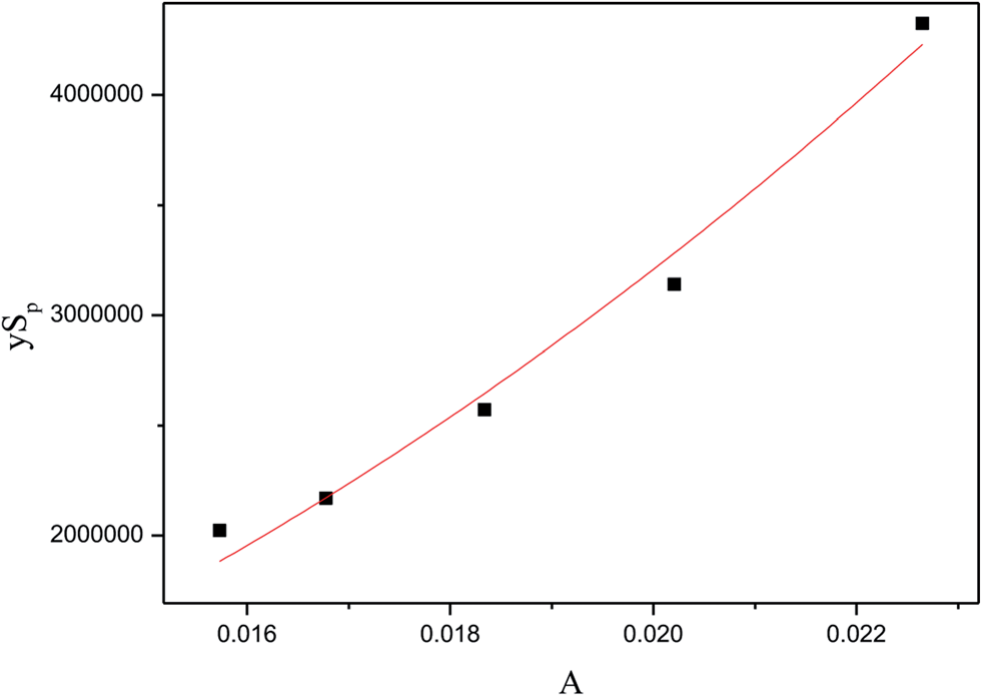
The relationship of pre-exponential factor (*A*) and membrane structure parameters (*yS*_p_).

Therefore, as shown in Fig. 8, the kinetics parameters could be obtained. The kinetic equation of inulin hydrolysis by membrane reactor was obtained as follows.17
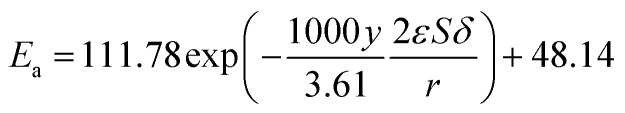
18
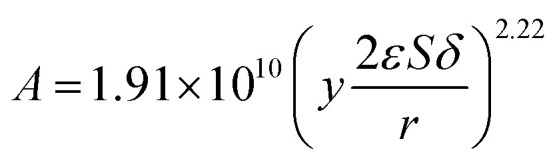


### 3.7. Model validation

**Fig. 9:**
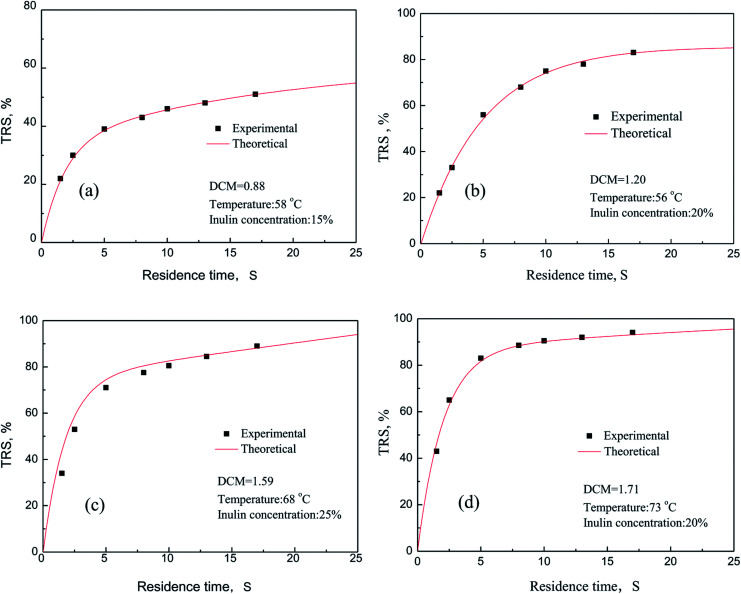
Reducing sugars yield curve as a function of process time.

The comparison of the experimental data with the theoretical prediction of the model was conducted. The results were shown in Fig. 9. In this comparison, a low value of relative error demonstrates the reasonable agreement between experimental and predicted value of the model. It is clear that all the experimental data fit reasonably well with the kinetic values under different reaction conditions. Meanwhile, the average error can be calculated in two groups randomly under the experimental conditions are obtained, respectively 1.3% and 6.9%. Among them, as seen in Fig. 9(c), theoretical value is slightly higher than the experimental results. One hand, inulin cannot be completely dissolved to the water medium because of the higher inulin concentration, and will increase the molecular diffusion resistance, which is not conducive to mass transfer. On the other hand, when the concentration of inulin is higher, the resistance of the reducing sugar released into the aqueous medium increases, which leads to the bigger average variances between the experimental results and theoretical values. In summary, the dynamics model for the PSF-ILs catalytic hydrolysis of inulin we established can be used to predict the catalytic performance of the catalyst, and the theoretical and experimental results validate the reliability of established kinetic model.

## 4. Conclusion

A novel PSF-ILs membrane endowed with catalytic action were first designed and prepared as a heterogeneous catalyst to produce reducing sugar by biomass hydrolysis in a catalytic membrane reactor. The optimal casting conditions with the highest catalytic efficiency of PSF-ILs membrane are 15.8% PSF-ILs, 14% additives (PVP+PEG600) and 70.2% DMF with the coagulation temperature of 45 °C, and the maximum TRS yields was up to 100% after two rounds inulin hydrolysis. A hydrolysis kinetic model involving membrane structure, such as membrane thickness, pore size, porosity and specific surface area, was established to predict inulin conversion. Meanwhile, the hydrolysis kinetics was studied under different conditions. In addition, the conversions obtained from the established kinetic model are in good agreement with the experimental data and could be also applied to predict the inulin hydrolysis in wider ranges of experimental conditions. Understanding the structure–property relationship of PSF-ILs membrane will be helpful to design the physical structure of membrane to improve its catalytic activity and reusability. This kind of novel green catalytic PSF-ILs membrane has potential application value in many catalysis fields.

## Conflicts of interest

There are no conflicts to declare.

## Supplementary Material

RA-008-C8RA00658J-s001
