# Supplementary material for: Kinetic model of biomass hydrolysis by a polysulfone membrane with chemically linked acidic ionic liquids via catalytic reactor
Source: RSC Adv. 2018 Mar 27;8(21):11714–24. doi: 10.1039/c8ra00658j (PMC9079156; doi:10.1039/c8ra00658j)
Supplement: RA-008-C8RA00658J-s001 [file RA-008-C8RA00658J-s001.pdf]

Electronic Supplementary Material (ESI) for RSC Advances.
This journal is © The Royal Society of Chemistry 2018

Kinetic model of the biomass hydrolysis by polysulfone membrane with chemically linked acidic ionic liquids via catalytic reactor

Peng Lu*, Yong Cao, Xiao-Lan Wang

2.2 Characterization

2.2.1 Fourier Transform Infrared (FT-IR)

FT-IR analysis of the PSF-ILs membrane was conducted in order to demonstrate the chemical structure of the membrane, the spectra of PSF(A), CMPSF(B) and PSF-ILs (C) membrane were shown in Fig. S1, the peaks at 3022 cm^{-1} of PSF membrane was due to the C-H stretching vibration of aromatic ring. Comparing with PSF(A), two peaks at 2972 cm^{-1} and 2855 cm^{-1} occurred in PSF-Cl(B), corresponding to the C-H asymmetric and symmetric stretching vibration of methylene. For PSF-ILs, the characteristic peaks C-H, C=C and C=N stretching vibration of imidazole ring were observed at 3044 cm^{-1}, 1711 cm^{-1} and 1685 cm^{-1} respectively, indicating that imidazole was successfully grafted on the PSF-Cl powder. In addition, the peaks at 1146 cm^{-1} and 1051 cm^{-1} were ascribed to the S=O asymmetric and symmetric stretching vibration of $-SO_3H$ group. All of the above observation indicated that the ILs had been covalently tethered to the PSF.

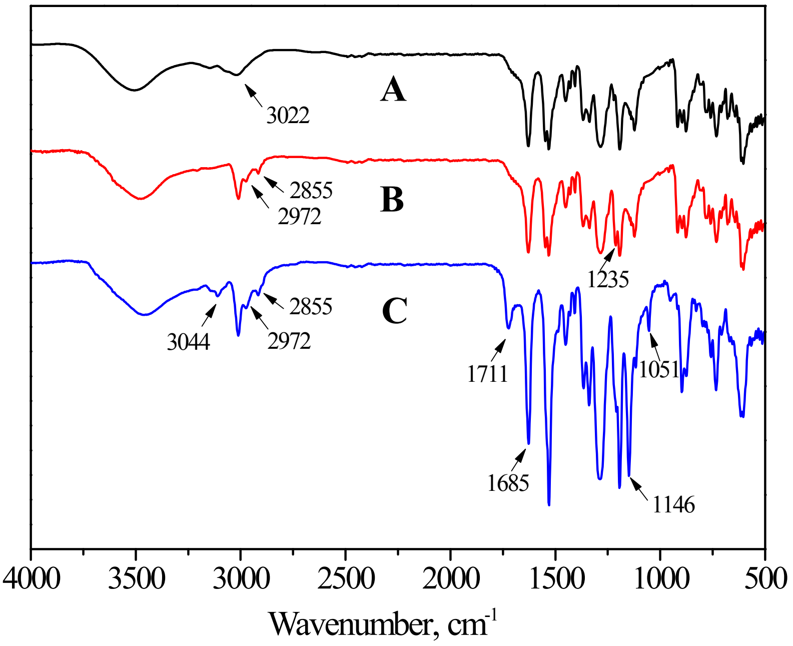

Fig. S1. FT-IR spectra of PSF(A), CMPSF(B) and PSF-ILs(C).

2.2.2 Nuclear Magnetic Resonance (^1H NMR)

^1H NMR spectroscopy was employed to determine the chemical structures of the prepared PSF-ILs membrane and the results were shown in Fig. S2. Comparing with spectrum A (PSF), the resonance at around δ 4.6 of the spectrum B (PSF-Cl) was assigned to the -CH_2Cl group in benzyl, which is in a close agreement with reported study. For spectrum C (PSF-ILs), the chemical shift of hydrogen atoms in -CH_2Cl group of PSF-ILs (δ=5.3) was higher than that of the CMPSF (δ=4.6), indicating the complete conversion of chloromethyl groups. Meanwhile, the signals arose at δ 7.8 and 9.2, which are ascribed to the protons of the imidazolium cations. In addition, the presented different peaks mainly ranging from δ 2.0-3.5 clearly confirmed the ring-opening reaction of the PSF-Im with the 1,4-butane sultone. Therefore, the results of ^1H NMR characterizations indicated that the ILs ([SO_3H-PMIM][HSO_4]) was immobilized onto the PSF.

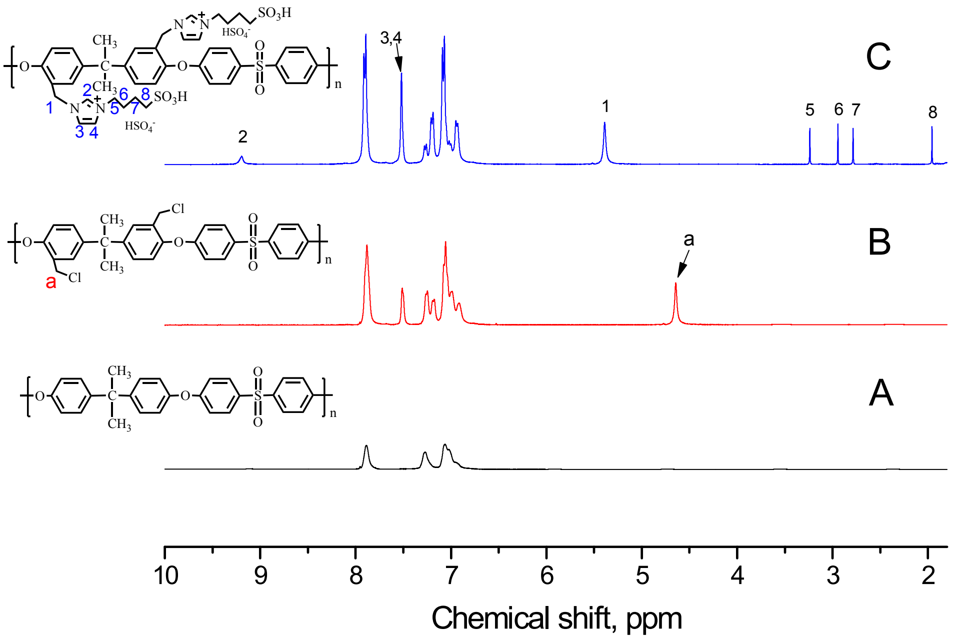

Fig. S2. ^{1}H NMR spectra of PSF(A), CMPSF(B) and PSF-ILs(C).

3.6 Kinetic study

3.6.1 Effect of membrane thickness on inulin hydrolysis

The thickness of the prepared membrane could be controlled by adjusting the thickness of the casting solution. Fig.S3 showed the variation of TRS with the increase the membrane thickness from 203 μm to 272 μm. TRS were found to grow with the increase of membrane thickness. This variation could be explained as: the residence time of substrate in the membrane increased with the enhancing of the membrane thickness during the membrane catalysis in filtration operation. The longer residence time would increase the collision probability between the active sites of ILs and the glycosidic bonds of inulin polysaccharides, and the more glycosidic linkages were hydrolyzed. Therefore, we can increase the thickness of the membrane as much as possible in case we desire. The experimental results showed that the TRS yield of 100% could be obtained after two rounds of hydrolysis by the membrane of 248 μm.

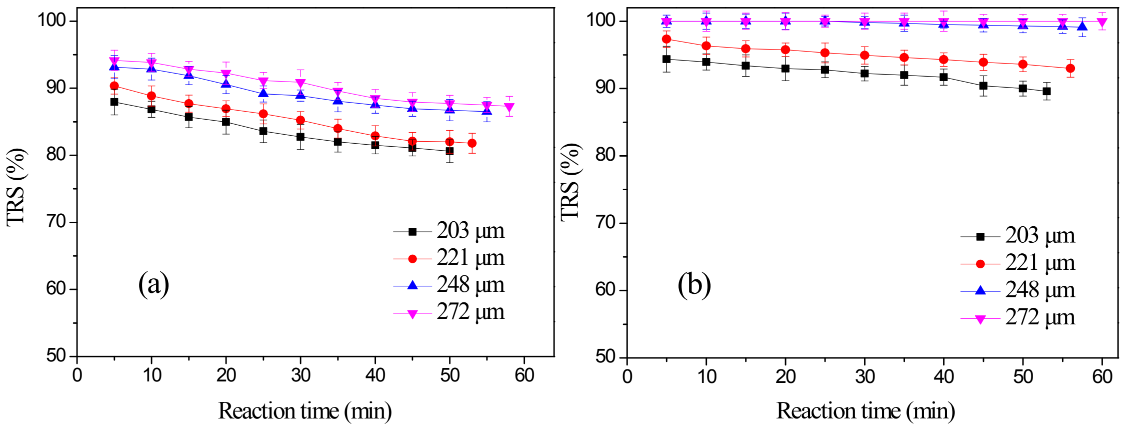

Fig.S3. Effect of the membrane thickness on yields of TRS. (a) First round hydrolysis, (b) Second round hydrolysis. Hydrolysis conditions: membrane flux 49.4 L/m^2·h, reaction temperature 65 ^{o}C.

3.6.2 Effect of porosity on inulin hydrolysis

In order to explore the effects internal structure of PSF-ILs membrane on the catalysis performance in more detail, we performed catalysis experiments employing the membranes with various porosities. The corresponding results were shown in Fig.S4. It is obvious that the TRS increased as the porosity of PSF-ILs membrane was increased. For the first round of hydrolysis, the conversion to reducing sugar increased from 76.1 to 90.2% with increasing porosity from 60.3 to 82.5%. For the latter two groups, the conversion could reach 100% after the second round hydrolysis. The reason seems due to the provided more effective internal pore wall for immobilizing the ILs, resulting in the higher conversion. Furthermore, when the porosity was greater than 77.6%, there is no obvious increase in conversion of reducing sugar. And considering economy, a higher initial inulin polysaccharide concentration is more favorable for fermentation solution, since it is desired to get higher ethanol concentration and then decrease the cost of ethanol/water separation in industry application, Hence, the appropriate porosity should be greater than 77.6% in the inulin hydrolysis system.

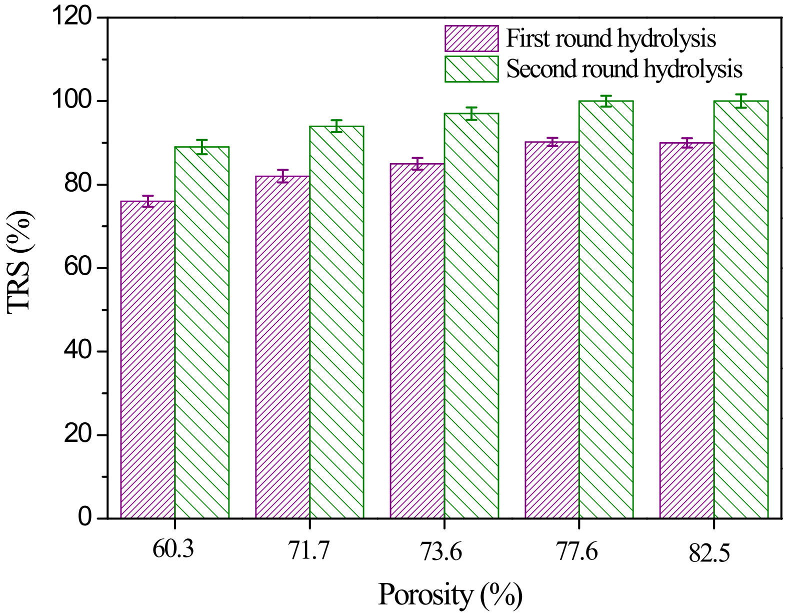

Fig.S4. Effect of the porosity on yields of TRS. Hydrolysis conditions: membrane flux 49.4 $L/m^2 \cdot h$, temperature 65 ^oC, membrane thickness 248μm.

3.6.3 Effect of pore size on inulin hydrolysis

Pore size was also an important parameter for the PSF-IL catalytic membrane. Fig.S5 depicted the variation of the TRS with an increase in pore size of PSF-IL membrane. For the first round of hydrolysis, with the increase of pore size from 22.1 to 72.5 nm, the corresponding TRS yields increased from 76.0 to 90.2%. These five kinds of catalytic membranes with different pore sizes exhibited better hydrolysis performance with yields up to 97% after the second round of hydrolysis. The results suggested that the increment of pore size is apparently favorable to accelerate the inulin hydrolysis. However, when pore size of PSF-IL membrane was above 56 nm, the conversion had no marked further change. Hence, the appropriate pore size of 56 nm was recommended.

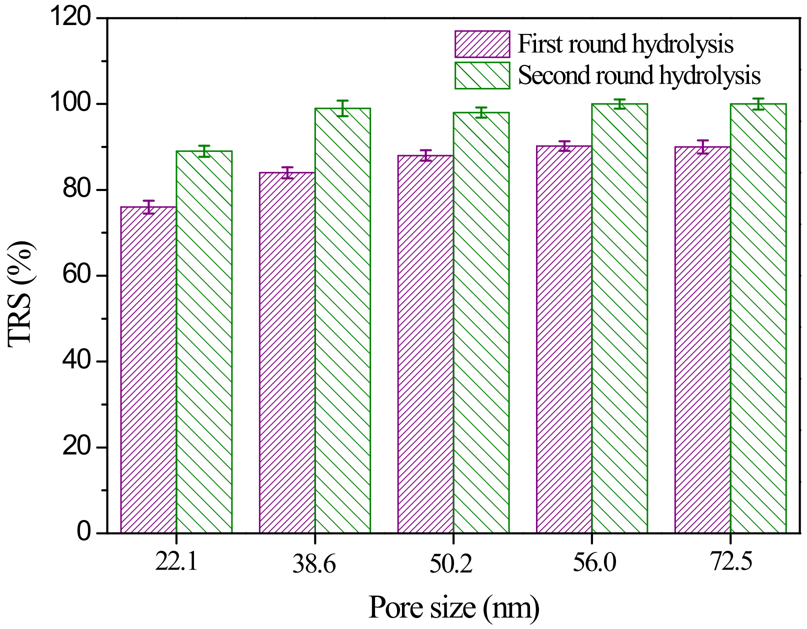

Fig.S5. Effect of the pore size on yields of TRS. Hydrolysis conditions: membrane flux 49.4 L/m^2·h, temperature 65 ^{o}C, membrane thickness 248 μm.
